# Increased immunogenicity of surviving tumor cells enables cooperation between liposomal doxorubicin and IL-18

**DOI:** 10.1186/1479-5876-7-104

**Published:** 2009-12-10

**Authors:** Ioannis Alagkiozidis, Andrea Facciabene, Carmine Carpenito, Fabian Benencia, Zdenka Jonak, Sarah Adams, Richard G Carroll, Phyllis A Gimotty, Rachel Hammond, Gwen-äel Danet-Desnoyers, Carl H June, Daniel J Powell, George Coukos

**Affiliations:** 1 Department of Obstetrics and Gynecology, University of Pennsylvania School of Medicine, Philadelphia, Pennsylvania, USA; 2 Abramson Family Cancer Research Institute, University of Pennsylvania School of Medicine, Philadelphia, Pennsylvania, USA; 3 Department of Pathology and Laboratory Medicine, University of Pennsylvania School of Medicine, Philadelphia, Pennsylvania, USA; 4 Division of Hematology-Oncology, University of Pennsylvania School of Medicine, Philadelphia, Pennsylvania, USA; 5 Department of Biostatistics and Epidemiology, University of Pennsylvania School of Medicine, Philadelphia, Pennsylvania, USA; 6 GlaxoSmithKline, Biopharm-CEDD, Biology US, King of Prussia, Pennsylvania, USA

## Abstract

**Background:**

Liposomal doxorubicin (Doxil) is a cytotoxic chemotherapy drug with a favorable hematologic toxicity profile. Its active drug, doxorubicin, has interesting immunomodulatory properties. Here, the effects of Doxil on surviving tumor cell immunophenotype were investigated.

**Methods:**

Using ID8 murine ovarian cancer cells, the immunomodulatory effects of Doxil were studied by measuring its impact on ovarian cancer cell expression of MHC class-I and Fas, and susceptibility to immune attack *in vitro*. To evaluate the ability of Doxil to cooperate with cancer immunotherapy, the interaction between Doxil and Interleukin 18 (IL-18), a pleiotropic immunostimulatory cytokine, was investigated *in vivo *in mice bearing ID8-Vegf tumors.

**Results:**

While Doxil killed ID8 tumor cells in a dose-dependent manner, tumor cells escaping Doxil-induced apoptosis upregulated surface expression of MHC-I and Fas, and were sensitized to CTL killing and Fas-mediated death *in vitro*. We therefore tested the hypothesis that the combination of immunotherapy with Doxil provides positive interactions. Combination IL-18 and Doxil significantly suppressed tumor growth compared with either monotherapy *in vivo *and uniquely resulted in complete tumor regression and long term antitumor protection in a significant proportion of mice.

**Conclusion:**

These data demonstrate that Doxil favorably changes the immunophenotype of a large fraction of the tumor that escapes direct killing thus creating an opportunity to expand tumor killing by immunotherapy, which can be capitalized through addition of IL-18 *in vivo*.

## Background

Successful cancer chemotherapy relies on the comprehensive tumor cell elimination. However, at clinically tolerated doses, chemotherapeutic drugs usually fail to kill all tumor cells *in vivo*. Theoretically, to achieve complete eradication, partial tumor killing by chemotherapy should be accompanied by a "bystander effect" in which the immune system recognizes, attacks, and eradicates residual tumor cells. Unfortunately, most cytotoxic anticancer agents used in the clinic exert immunosuppressive side effects.

Doxorubicin (or adriamycin) is an anthracycline antibiotic that intercalates with DNA, inhibiting its replication. Pegylated liposomal doxorubicin (Doxil) extravasates efficiently through the leaky tumor vasculature and is protected from renal clearance, enzymatic degradation, and immune recognition, enhancing drug pharmacokinetics, reducing hematologic effects and achieving targeted delivery to the tumor site. Unlike other chemotherapeutic agents, Doxorubicin possesses interesting immunomodulatory properties, potentiating Her-2 cancer vaccination in mice [1] and inducing immunogenic tumor cell apoptosis [2,3]. Tumors are however known to escape immune attack through downregulation of surface molecules that mediate antigen presentation and immune recognition, such as major histocompatibility complex (MHC) molecules, and modulating death receptors and other immunomodulatory ligands. Accordingly, investigation is required to elucidate mechanisms that both increase the immunogenicity of tumor cells surviving chemotherapy and boost effector immune mechanisms.

Immunostimulatory cytokine therapy may be an attractive approach to capitalize on the immune effects of doxorubicin. Doxorubicin has been shown to enhance the therapeutic effect of TNF-α, IL-2 and IL-12 in mouse models of cancer [4-6]. Interleukin-18 (IL-18) has now emerged as a novel cytokine with potent immunostimulatory properties which affects multiple subpopulations of immune cells of the adaptive and innate immune system. It activates effector T cells; induces IFN-γ, TNF-α, IL-1α, and GM-CSF production; promotes Th1 differentiation of naive T cells; and augments natural killer (NK) cell cytotoxicity [7-10]. IL-18 promotes protection against tumor challenge in mice [7]. In phase I evaluation, recombinant human (rh)IL-18 monotherapy has been safely administered to 28 patients with solid tumors, with two partial tumor responses [9]. Compared with other immunostimulatory cytokines, its toxicity profile is remarkable; mild to moderate toxicities even with repeat administration and a maximum tolerated dose that has not been reached [11]. IL-18 enhanced activation of peripheral blood CD8^+ ^T cells, NK cells and monocytes and induced a transient increase in Fas ligand (FasL) by circulating CD8^+ ^T cells and NK cells [11].

We hypothesized that IL-18 a well suited drug for combinatorial therapies with pegylated Doxil to enhance clinical efficacy. Doxil has become standard second line drug for the treatment of patients with platinum refractory or resistant disease ovarian cancer. Importantly, cell-mediated immune mechanisms appear to play a role in controlling progression of ovarian carcinoma [12] and early phase clinical results suggest that the use of immunotherapy can provide clinical benefit in ovarian cancer [13]. Because the effect of immune therapy becomes clinically relevant only if immune mechanisms target the tumor fraction surviving chemotherapy, we studied the fate of tumor cells escaping direct killing by Doxil. We hypothesized that tumor surviving Doxil chemotherapy becomes sensitized to cytotoxic lymphocytes and can be effectively targeted by the immune response activated by IL-18, providing the basis for positive therapeutic interactions.

## Materials and methods

### Cell culture

ID8 ovarian cancer cells were donated by Drs. Kathy Robby and Paul Terranova (Kansas University)[14]. ID8-Vegf and ID8-E6E7 cell lines were described elsewhere [15,16]. ID8, ID8-E6E7 and ID8-Vegf cells were maintained in DMEM media (Invitrogen, Carlsbad, CA) supplemented with 10% fetal bovine serum (FBS), 100 U/ml penicillin, and 100 μg/ml streptomycin (Roche, Indianapolis, IN) in 5% CO_2 _at 37°C.

### Mice

Eight week old female C57BL/6 mice (Charles River Laboratories, Wilmington, MA) were used in protocols approved by the Institutional Review Board of the University of Pennsylvania.

### Tumor inoculation

For intraperitoneal (i.p.) tumors, ID8-Vegf cells were injected at 5 × 10^6 ^per mouse. For subcutaneous (s.c.) tumors, a single cell suspension of ID8-Vegf cells was prepared in phosphate buffered saline (PBS) mixed with an equal volume of cold Matrigel. 10^7 ^cells in 0.5 ml total volume was injected into the flank. Tumors were detectable two weeks later. Tumor size was measured weekly using a Vernier caliper. Tumor volumes were calculated by the formula V = 1/2 (L × W)^2^, where L is length (longest dimension) and W is width (shortest dimension). When control tumors reached the size of ~800 mm^3^, animals were sacrificed, and tumors excised and weighed.

### *In Vivo *Treatment

Mice were treated with i.p. bolus injections of Doxil in the range of 17% to 50% of maximally tolerated dose (MTD) for mice [17] or 5% dextrose weekly for 4 weeks. Chemotherapy treatment started one week (i.p. model) or 14 days (s.c. model) after tumor inoculation; IL-18 treatment began 2 days later. IL-18 or 0.9% saline was given s.c. at 10, 30 or 100 μg/mouse, daily for 50 days.

### *In vitro *treatment of tumor cells

ID8 cells were exposed to Doxil at 0, 0.1, 0.3 or 1 μg/ml concentrations for 6 hours. The cells were washed twice with PBS, and cultured in drug-free media for another 42 hours. ID8 cells were then washed twice with PBS, trypsinized and counted. Non-viable cells were excluded using Trypan Blue staining. Fas-induced killing was mediated by anti-Fas agonistic monoclonal antibody (mAb) Jo2 (BD PharMingen) crosslinked using Protein G (2 μg/ml; Biovision) or isotype-matched Ab and protein G. Antibody was added 24 hours before cell harvesting and counting.

### Flow cytometry

Cells were blocked and stained with biotinylated anti-MHC-I (H-2K^b^/H-2D^b^) mAb with APC-labeled Streptavidin, PE-labeled anti-Fas mAb or isotype-matched controls (BD PharMingen, San Diego, CA). Apoptosis was measured using TACS Annexin V-FITC apoptosis detection system (R&D Systems; Minneapolis, MN). Analysis was performed using a FACS Canto cytometer.

### Cytotoxicity assay

ID8-E6E7 cells were used as targets in a colorimetric non-radioactive cytotoxicity assay measuring LDH (Promega). Target cells (12 × 10^3 ^cells/well) were coincubated with T cells at various E:T cell ratios, in 200 μl RPMI-10 (RPMI supplemented with 10% FBS, 100 U/ml Penicillin, and 100 ug/ml Streptomycin) for 4 hrs at 37°C in 5% CO_2_. Effector cells were from eight to sixteen-week old C57BL/6 mice vaccinated twice, one week apart, with DNA plasmid vaccine encoding the E7 peptide and Listeriolysin O as an adjuvant, kindly provided by Dr. Yvonne Paterson. One month later, mice were inoculated s.c. in the flank with 50,000 E7 expressing TC-1 cells. Two weeks later mice were sacrificed; splenocytes isolated; and stimulated in vitro for 7 days with 8 μg/ml E7 peptide and 30 IU/ml IL-2 in RPMI-10. % specific cytotoxicity = (experimental - spontaneous/maximum - spontaneous) × 100.

### Statistical analysis

Two-tailed Student's t-test was used for between-group comparisons with *in vitro *and flow cytometry data. Differences between treatment groups were considered significant at the level of p < 0.05. Kaplan-Meier survival curves were computed. A Cox regression model was used to obtain the hazard ratios (HR) for each treatment group compared to the control group and their 95% confidence intervals.

## Results

### Doxil treatment favorably alters cancer cell immunophenotype in vitro

**Figure 1:**
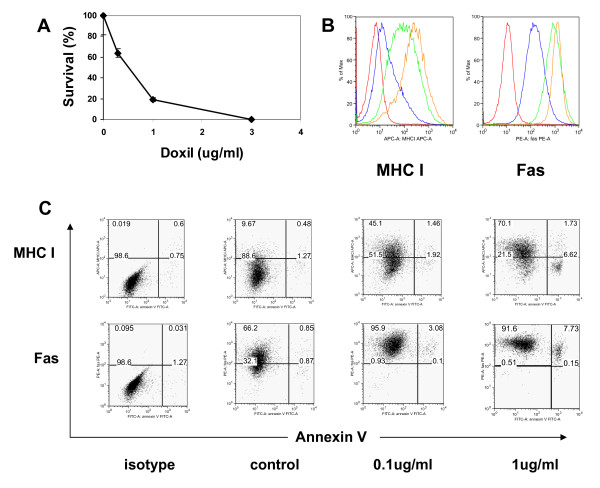
**Doxil treated ovarian cancer cells upregulate MHC class-I and Fas expression in vitro**. **(A) **ID8 cells were exposed to titered concentrations of Doxil (0, 0.3, 1 and 3 ug/ml) and measured for viable cell countsmeasured. ID8 cells were either incubated in culture media alone or with the indicated concentration of Doxil for 6 hours, washed, and incubated in drug-free media for 42 hours before harvesting. **(B) **Upregulation of MHC-I (left) and Fas (right) on ID8 cells following treatment with Doxil and staining with MHC-I and Fas antibodies. Histogram: Isotype control (red); untreated (blue); Doxil 0.1 μg/ml (green); Doxil 1 μg/ml (brown). All the histograms depict Annexin-V negative (non apoptotic cells). **B) **Dot plot diagrams depict the upregulation of MHC-I and Fas in gated non-apoptotic (Annexin v-negative) tumor cells exposed to Doxil 42 hours before.

Cell damage induced by chemotherapy can sensitize tumor to immune effector cells [18]. To assess the capacity of Doxil to sensitize ovarian cancer cells to immune attack, we identified doses of Doxil *in vitro *at which greater than 50% of ID8 cells remained viable (Figure 1A). ID8 cells were exposed to Doxil for 6 hours, washed and incubated for an additional 42 hours in drug-free media. At concentrations ≤ 0.3 ug/ml, Doxil reproducibly yielded cell cultures with cell viability > 50% (Figure 1A). Treated ID8 cells were harvested and analyzed for cell surface phenotype by flow cytometry. Doxil induced a significant, dose-dependent upregulation of MHC-I and Fas in ID8 tumor cells (Figure 1B). There was no significant increase in the expression of MHC-II, the NKG2D ligands RaeI and H60 or death receptors 4 (DR4) and DR5 in ID8 cells following exposure to Doxil (data not shown).

Chemotherapeutic agents can promote MHC-I or NKG2D ligand upregulation by tumor cells and to sensitize them to Fas or TRAIL mediated apoptosis [19,20], but it is unclear whether this occurs mainly in tumor cells destined to die from chemotherapy-induced cytotoxicity or the fraction of cells surviving the chemotherapeutic insult. We found that Doxil induced a significantly upregulated MHC-I and Fas in non-apoptotic (Annexin V-negative) ID8 tumor cells (Figure 1C). At the 1 μg/mL Doxil concentration, the majority (> 75%) of the non-apoptotic cells upregulated MHC-I, compared to less than 10% in the untreated group. Fas expression was detectable at an intermediate level in untreated cells, but was expressed at high levels on all ID8 viable cells at both 0.1 and 1 μg/mL Doxil concentrations, with higher expression levels at the increased drug concentration.

### Doxil treated cancer cell are more susceptible to immune attack

**Figure 2:**
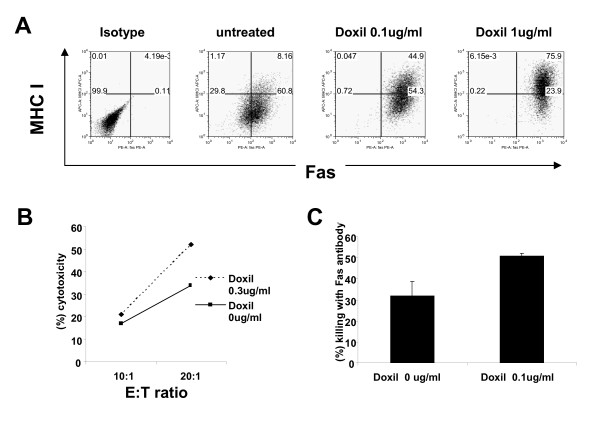
**Increased susceptibility of viable ovarian cancer cells to immune attack after Doxil exposure**. **(A) **Upregulation of MHC-I and Fas on ID8-E6E7 cells following treatment with Doxil at 0.1 μg/ml or 1 μg/ml. **(B)** Left, Increased sensitivity of ID8-E6E7 cells to CTL activated with IL-2 and E7 peptide. The effector to target ratio (E:T) is indicated. Each data point represents the mean of triplicate wells. Experiments repeated twice with similar results. **(C) **Treatment of ID8 cells with Doxil sensitizes them to Fas agonistic antibody (right). The ID8 cells (untreated or treated with Doxil) have been incubated with the Fas agonistic antibody and recombinant protein G or with isotype matched antibody and recombinant protein G for 24 hours. Cells were harvested (trypsin), stained with trypan blue and viable cells were counted. The bars show the means and standard error of the mean for three independent experiments.

Increased expression of immune-associated molecules by viable ID8 cells following Doxil exposure suggested their elevated susceptibility to immune recognition and killing. To test this hypothesis, ID8-E6/E7 cells, expressing human papilloma virus E6 and E7 as surrogate tumor antigens [16], were exposed to Doxil for 6 hrs at 1 μg/ml. (Figure 2A). Forty-two hrs later, the majority of viable ID8-E6/E7 tumor cells co-expressed MHC-I and Fas, similar to the ID8 control line (Figure 2A). E7-reactive CD8 effector T cells harvested from E7-vaccinated mice and stimulated *in vitro *using synthetic E7 peptide were coincubated with ID8-E6/E7 and control ID8 target cells that had been exposed to Doxil for 6 hrs. Doxil exposure increased the susceptibility of ID8-E6/E7 target cells to T cell-mediated lysis at a 20:1 ratio compared to untreated ID8-E6/E7 controls (Figure 2B) or control ID8 cells (not shown). To evaluate the susceptibility of ID8 cancer cells surviving Doxil to Fas-mediated cell death, Doxil-treated and untreated ID8 cells were incubated with Fas agonistic antibody or with isotype matched antibody for 24 hours, and measured for viability. ID8 cells exposed to Doxil also showed increased sensitivity to Fas agonistic antibody (two-tailed t-Test; p = 0.002; Figure 2C).

### Positive interaction between Doxil and IL-18 immunotherapy in vivo

**Figure 3:**
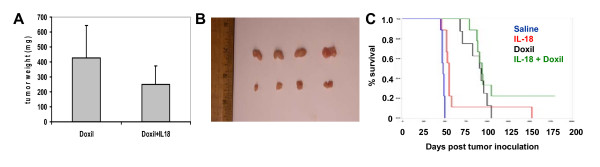
**Combination therapy for C57BL/6 mice injected in the flank with ID8-Vegf cells**. Tumors from mice treated with Doxil with or without IL-18 were excised and weighed when the Doxil treated tumors reached the size of 600 mm^3^. Results are medians (50^th ^percentile); error bars: interquartile range (25%-75%), (n = 9). **(A) **The Doxil-IL-18 combination treatment restricts significantly the tumor weight compared to the Doxil treated group (p = 0.034) (upper graph). Doxil was given at 4 mg/kg/dose for 4 weekly doses starting two weeks after tumor inoculation, while IL-18 was given at 10 μg daily for 50 days starting two days later. **(B) **The picture shows four tumors from mice treated with the combination of IL-18 and Doxil (upper row) and four tumors from mice treated with Doxil monotherapy (lower row). **(C) **The effect of mono- and combination therapy on tumor growth *in vivo*. C57BL/6 mice were injected i.p. with ID8-Vegf cells and subsequently treated. The chemotherapy treatment was started one week after the tumor challenge and IL-18 treatment 2 days later. In the Doxil-IL-18 combination group, 22% of the mice remained tumor-free 6 months after the tumor challenge while in the groups treated with either monotherapy the overall 6-month survival was 0% (untreated control: n = 9, IL-18: n = 9, Doxil: n = 8, Combination: n = 9). The tumor-free mice were rechallenged with ID8-Vegf cells injected s.c. and the tumors were rejected.

Sensitization of ID8 tumor cells by Doxil to cytotoxic T cell-mediated lysis suggested that immunostimulatory cytokine therapy could effectively target chemotherapy-surviving cancer cells and in combination improve the efficacy of Doxil therapy. We therefore tested IL-18 and Doxil combination therapy in C57BL/6 mice inoculated s.c. with ID8-Vegf tumors. Compared to Doxil monotherapy, combinatorial therapy significantly decreased tumor growth (Figure 3A and 3B). Median tumor weight and interquartile range was 400 mg (271.5-604) in the Doxil treatment group and 220 mg (190-280; Student's t-Test, p = 0.034) in the combinatorial treatment group (Figure 3A).

Both monotherapies and combination therapy significantly improved survival compared to the untreated control group (IL-18 group, p < 0.001; Doxil group, p < 0.001; log-rank test) (Figure 3C). Median survival was increased in mice receiving Doxil therapy (with or without IL-18) compared to IL-18 monotherapy. Median survival was similar in mice receiving Doxil therapy with or without IL-18; however tumor cures were only observed in mice receiving combinatorial therapy. Combination Doxil/IL-18 therapy resulted in 22% 6-month overall survival compared to 0% for the respective monotherapies (Figure 3C). All tumor-cured animals were effectively protected from s.c. re-challenge with ID8-Vegf cells.

**Figure 4:**
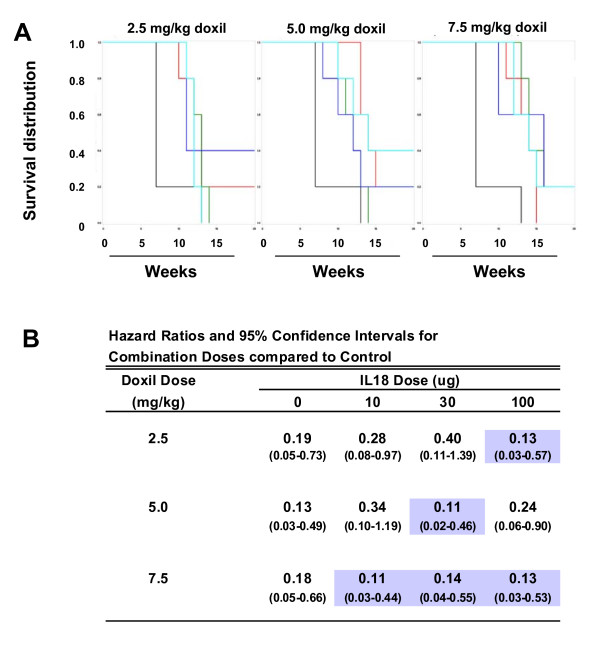
**Combining different doses of Doxil and IL-18 for optimized therapy**. **(A) **Kaplan-Meier survival curves show the effects of Doxil therapy at 2.5, 5, or 7.5 mg/Kg when combined with different doses of IL-18 and administered to ID8-Vegf tumor bearing mice (n = 5/dose group). Mice received IL-18 doses of 10 (green), 30 (teal), or 100 (blue) ug/mouse, or no IL-18 as control (red). Mice were treated with i.p. bolus injections of Doxil or 5% dextrose control (black) given weekly for 4 weeks. Chemotherapy treatment started one week after i.p. tumor inoculation; IL-18 treatment began 2 days later. IL-18 or 0.9% saline was given s.c. at 10, 30 or 100 μg/mouse, daily for 50 days. **(B) **Improved survival was determined in combination therapy groups using the hazard ratios (HR) for each treatment group compared to the control group and their 95% confidence intervals. Combinations of IL-18 and Doxil showing improved survival HR are shaded.

To optimize dosing, we combined different doses of Doxil (2.5, 5 or 7.5 mg/kg) with different doses of IL-18 (10, 30 or 100 μg) and computed Kaplan-Meier survival curves (Figure 4). Untreated mice died in less than 15 weeks after tumor inoculation. Compared to the control mice, at the lowest dose of Doxil (2.5 mg/kg), the most significant improvement in survival was at the 100 μg dose of IL-18 (HR = 0.13, 95% confidence interval of 0.03-0.57). At the intermediate Doxil dose of 5 mg/kg, the most significant improvement in survival was with IL-18 at the intermediate (30 μg) dose (HR = 0.11, 0.02-0.46). At the highest Doxil dose (7.5 mg/kg), there was improved survival at all three doses of IL-18 (HR = 0.11, 0.14, 0.13, respectively). The combination of Doxil at 5 mg/kg with IL-18 at 30 μg resulted in a 6-month survival of 40%. A similar level of survival was obtained with combination Doxil at 2.5 mg/kg with IL-18 at 100 μg (Figure 4) suggesting that the efficacy of Doxil therapy for ovarian cancer can be improved by the addition of IL-18.

## Discussion

The identification of favorable chemotherapy and immune therapy combinations remains a critical task for improving cancer outcomes. Doxil has not been reported to exhibit T cell suppressive activity to date and its low hematologic toxicity profile makes it an ideal drug to combine with immunotherapy. Our findings show that tumor cells surviving Doxil upregulate surface molecules that are critical for immune recognition and attack such as MHC class I and Fas through an unknown mechanism, and exhibit increased sensitivity to killing by cytotoxic lymphocytes and to apoptosis mediated by Fas in vitro. Therefore, in addition to direct tumor killing and the immunization effect derived from immunogenic cell death, Doxil exerts an important immunomodulatory effect upon the tumor fraction surviving drug exposure. This effect is distinct and complementary to the previously described effect of adriamycin which was shown to elicit a vaccination effect by mediating immunogenic death in tumor cells. Anthracycline-induced immunogenic death is associated with caspase activation [2] and mediated by rapid, preapoptotic translocation of calreticulin to the cell surface, promoting immunogenicity [3]. The effect observed in our studies is indeed distinct as it affects primarily the non-apoptotic fraction of tumor following treatment with Doxil.

The combination of IL-18 with Doxil at doses below the maximally tolerated dose substantially restricted tumor growth in comparison with Doxil or IL-18 monotherapy. Improved tumor control by combination therapy is presumed to be mediated through the amalgamation of IL-18 mediated immune activation, with Doxil-mediated tumoricidal activity and increased immunogenicity of the surviving tumor cell fraction in vivo. Although upregulation of MHC class I and Fas expression by surviving tumor cells was not evaluated on tumor biopsies, the combinatorial therapy produced complete tumor regression and cure in a substantial number of mice, while no cures were observed in mice treated with pegylated liposomal doxorubicin or IL-18 monotherapy. Thus, the addition of IL-18, an immunostimulatory cytokine with an established safety profile, to standard Doxil chemotherapy may significantly increase tumor response and lead to increased tumor elimination.

IL-18 has recently emerged as an immunostimulatory cytokine with the capacity to augment anticancer therapy. In mice, IL-18 promotes protection against tumor challenge, and enhances NK cell cytotoxocity and T cell effector function [7,10]. IL-18 has immunostimulatory effects on human cells as well. Administration of IL-18 has been shown to augment adoptive human T cell transfer in a xenogeneic mouse model of graft versus host disease, by diminishing the engraftment of regulatory T cells and enhancing the engraftment of effector T cells and pathology in vivo [21]. As a monotherapy, IL-18 achieved limited clinical efficacy in a phase I study, however, IL-18 did increase activation molecule expression on circulating T cells, NK cells and monocytes, and induced a transient increase in Fas ligand (FasL) expression by circulating CD8^+ ^T cells and NK cells [9,11]. Earlier reports also suggested a role for IL-18 as an anti-angiogenic inhibitor of solid tumor outgrowth [22]. Reciprocally, the immunostimulatory capacity of IL-18 may promote the aggressiveness of myeloid leukemia cells [23]. In ovarian cancer, IL-18 single nucleotide polymorphism (SNP) analysis does not reveal evidence for an association with epithelial ovarian cancer risk [24]. However, increased levels of serum IL-18 has been reported to correlate with advanced disease, which mechanistically may reflect production by tumor-stimulated immune cells or by tumor cells themselves [25]. Accordingly, the capacity to provide super-physiological concentrations of IL-18 through passive cytokine administration provides the opportunity to promote antitumor responses in patients with ovarian cancer in vivo.

The observed positive interaction between Doxil and IL-18 complements previous evidence that adriamycin can enhance immunotherapy [1,5]. However, in these prior studies, doxorubicin was administered prior to immunotherapy, making it possible that this effect was mediated by attenuation of immunosuppressive mechanisms. Our findings are unique in that they report long term co-administration of chemotherapy with cytokine therapy and suggest that this positive drug interaction results in significant expansion of the tumor fraction that is killed by the combined therapy. The favorable safety profiles of both IL-18 and Doxil, coupled with the potent therapeutic effect of their combination reported herein, warrants the clinical evaluation of this combinatorial approach in ovarian cancer.

## Conclusion

In conclusion, we provide evidence that Doxil favorably alters the immunophenotype of cancer cells that survive direct killing allowing for increased tumor killing by IL-18 immunotherapy *in vivo*.

## Abbreviations

MHC: major histocompatibility complex; IL-18: Interleukin 18; NK: natural killer; FasL: Fas ligand; MTD: maximally tolerated dose.

## Competing interests

The authors declare that they have no competing interests.

## Authors' contributions

IA, FB, SA carried out in vitro evaluation of Doxil impact on immunophenotype, and susceptibility to immune attack using the ID8 ovarian cancer cell line. AF, CC, GDD performed in vivo assessment of combinatorial therapy in tumor bearing mice and provided E7 peptide primed T cells for in vitro assays, GC, ZJ, RGC and CHJ provided key reagents and cell lines and guided study design, DJP and GC drafted the manuscript.

Conceived and designed the experiments: CHJ, GC. Performed the experiments: IA AF CC FB SA RGC GD. Analyzed the data: DJP PG RH IA. Contributed reagents/materials/analysis tools: ZLJ. Wrote the paper: DJP IA GC.

All authors have read and approved the final manuscript.
